# Contribution of Tocopherols in Commonly Consumed Foods to Estimated Tocopherol Intake in the Chinese Diet

**DOI:** 10.3389/fnut.2022.829091

**Published:** 2022-06-08

**Authors:** Yu Zhang, Xin Qi, Xueyan Wang, Xuefang Wang, Fei Ma, Li Yu, Jin Mao, Jun Jiang, Liangxiao Zhang, Peiwu Li

**Affiliations:** ^1^Oil Crops Research Institute, Chinese Academy of Agricultural Sciences, Wuhan, China; ^2^Key Laboratory of Biology and Genetic Improvement of Oil Crops, Ministry of Agriculture and Rural Affairs, Wuhan, China; ^3^Quality Inspection and Test Center for Oilseed Products, Ministry of Agriculture and Rural Affairs, Wuhan, China; ^4^Hubei Hongshan Laboratory, Wuhan, China

**Keywords:** China, vitamin E, vegetable oil, dietary intake, dietary supplementation

## Abstract

Vitamin E is an essential fat-soluble nutrient mainly found in vegetable oils, nuts, and other foods. In this study, we evaluated the contribution of commonly consumed foods to the vitamin E dietary intake of the population in relation to their consumption practices. In addition, the vitamin E intakes of Chinese residents were compared in different regions of China and in different years. The results showed that vegetable oil was the main source of vitamin E dietary intake for Chinese residents, accounting for 46.76% of total dietary intake of vitamin E, followed by cereals, vegetables, meat, aquatic products, eggs, legumes, nuts, fruits and dairy products. Among all vegetable oils, rapeseed oil was the highest contributor of vitamin E, accounting for 10.73% of all foods. Due to dietary habits and regional differences, vitamin E intake also varies greatly among residents in different regions of China and has increased yearly from 1982 to 2020. This study provides with scientific evidence for reasonable VE supplementation.

## Introduction

Vitamin E (VE) is an essential fat-soluble nutrient in numerous foods such as nuts, seeds and vegetable oils. As a natural antioxidant, VE could stop the production of reactive oxygen species (ROS) formed when fat undergoes oxidation. In addition, VE is of clinical importance for the modulation of immune function, as it affects the susceptibility of the host to infectious diseases (1). Moreover, VE was found to improve vascular function in diabetic patients (2). Together with vitamin C, VE significantly improved cognitive function in old mice (3). The previous showed that a moderate VE supplementation significantly protected sperm quality in males and egg quality in females by reducing lipid peroxidation in sperm (4). Dietary supplementation with 50–100 mg/kg vitamin E significantly improved the growth and intestinal performance of animals, increasing the activity of several digestive enzymes. Furthermore, VE supplementation significantly increased the height of the intestinal folds and the thickness of the mucosa (5). Theoretically, the functions of different tocopherols vary with their structure (6). For example, γ-tocopherol was effective in inhibiting colon and lung cancer, and controlling cancer progression, while δ-tocopherol was superior to α-tocopherol and γ-tocopherol in tumor-inhibiting activity (7). Moreover, α-tocopherol is also found to be a reflection of daily dietary status (8). All tocopherols except β-tocopherol inhibit smooth muscle proliferation, and α-tocopherol is also not the only isomer important for human health (9). More importantly, no adverse effect from consuming VE in food was reported. Only VE supplements at very high dose (>1,000 mg/day) might lead pro-oxidant damage (10).

Both plant-based diets and animal products are important sources of VE, with the amount varying in different foods. Nuts, seeds, and vegetable oils contain high levels of tocopherols, as do green leafy vegetables and fortified cereals (11). The dietary intake of VE varies from country to country, and also from time to time, due to different dietary structures and eating habits. To increase VE intake, many VE fortified products such as VE encapsulation appeared in market (12). However, it is not clear whether the body is deficient in vitamin E or not. Without dietary nutritional data, overconsumption of VE fortified products often occurs. Therefore, it is necessary to assess the dietary intake of the population to determine whether different groups of people lack VE while also providing scientific and reasonable guidance on VE supplementation.

Dietary assessment is an important tool for the scientific guidance of a diet. Dietary assessment mainly focuses on the comprehensive assessment of the dietary intake of individuals and provides assessment results. Among existing dietary assessment methods, food frequency questionnaires (FFQs) have been widely used in large epidemiological studies since the 1990s (13). After its accuracy was questioned in the early 2000s, many changes in assessment methods occurred. Some researchers shifted their attentions to improving the feasibility and accuracy of open-ended dietary assessment methods rather than improving the FFQ or further searching for relevant biomarkers, while other researchers focused on improving the accuracy of the FFQ (14). The dietary consumption and nutritional status of women undergoing chemotherapy were evaluated for breast cancer (15). In our previous study, the content and composition of phytosterols in different vegetable oils were analyzed to estimate the total intake of phytosterols and the contribution of food to nutrient intake based on consumption data (16). Three national surveys in the United States including the 2001–2002 National Health and Nutrition Examination Survey (NHANES), NHANES III (1988–1994), and the Continuing Survey of Food Intakes by Individuals (1994–1996) were conducted to assess the intake levels of vitamin E of the diets of most Americans (17). At present, the composition and determination of vitamin E among the important evaluation criteria of vegetable oils are less studied. In addition, there are fewer studies on the composition and determination of vitamin E. Therefore, the intake of vitamin E for the population is still unclear. Moreover, there is no systematic study to assess the intake of vitamin E in Chinese populations. So, it is necessary to conduct this dietary assessment.

In this study, the composition and content of vitamin E in main kinds of foods in Chinese daily diets were summarized, and the amount of VE intake was calculated. Moreover, the distribution in different regions and historical changes in the VE intake of Chinese residents were analyzed for reasonable diet and appropriate nutritional supplements.

## Materials and Methods

### Data Source

Data on the tocopherol content and composition in cereals, grains, potatoes, legumes, vegetables, fruits, nuts, vegetable oils, meat, eggs, milk, and aquatic products used in this article were obtained from the Chinese Food Composition Tables released by National Institute for Nutrition and Health, Chinese Center for Disease Control and Presentation. In this database, there are more than 3.3 thousand kinds of plant derived foods and more than 3.6 thousand kinds of animal derived foods. China's domestic consumptions of main kinds of foods were obtained from production, supply, and distribution (PSD) reports released by United States Department of Agriculture (USDA), China Statistical Yearbook, and China Population Nutrition and Health Status Monitoring Report. From the China Population Nutrition and Health Status Monitoring Report and USDA PSD reports, the consumption of major foods of Chinese residents, including cereals, potatoes, beans, vegetables, fruits, nuts, vegetable oil, meat, eggs, milk, and aquatic products. As shown in Supplementary Table 1, beans include soybeans, mung beans, and red beans, which account for the largest consumption proportion in China, while 26 vegetables are selected that Chinese residents consume the most and frequently. The source of fruit consumption in USDA PSD reports mainly includes apple, banana, pear, grape, peach, orange, orange, grapefruit, and cherry. Finally, taking the tocopherol content and consumption into consideration, as shown in Supplementary Tables 1, 2, 60 kinds of foods and 12 kinds of vegetable oils were selected to assess the estimated tocopherol intake in the Chinese diet.

### Calculation Method

Although the four chemical structures of tocopherols are similar, their biological activities are different. The body absorbs different tocopherols to different degrees. α-Tocopherol is the most widely distributed, while the activity of β-, γ-, and δ-tocopherol is 50, 10, and 2% of that of α-tocopherol, respectively. Therefore, the amount of VE in food is often expressed as α-tocopherol equivalent (α-TE) and could be calculated by the following conversion formula.


(1) $$\begin{array}{l} \text{α}-\text{TE }(\text{mg})\text{ = α}-\text{tocopherol }(\text{mg})+0.5\;\times \text{ β}-\text{tocopherol }(\text{mg})\\ \;+\;0.1\times \text{γ}-\text{tocopherol }(\text{mg})+0.01\\ \begin{array}{cc} \;\times & \text{δ}-\text{tocopherol }(\text{mg}) \end{array} \end{array}$$

Firstly, α-TE values of commonly consumed foods were calculated by the levels of tocopherols in various foods recorded in Chinese Food Composition Tables and the above formula, and shown in Supplementary Tables 1, 2. Then, the estimated VE intake from each food was calculated by product of α-TE values and the daily consumption of commonly consumed foods queried in the USDA and China Statistical Yearbook, respectively. Finally, the estimated tocopherol intake in the Chinese diet was calculated by sum of the estimated VE intakes from commonly consumed foods. Moreover, we also calculated the contribution of a certain kind of foods to total VE intake (16).

Moreover, although α-TE is commonly used to assess the VE content in foods, other isomers also have different functions and effects on an organism. For example, as abovementioned, δ-tocopherol was superior to α-tocopherol in tumor-inhibiting activity (7). Thus, we also assessed the estimated VE intake calculated by product of the total amount of tocopherols and the daily consumption of commonly consumed foods queried in the USDA and China Statistical Yearbook.

**Figure 1:**
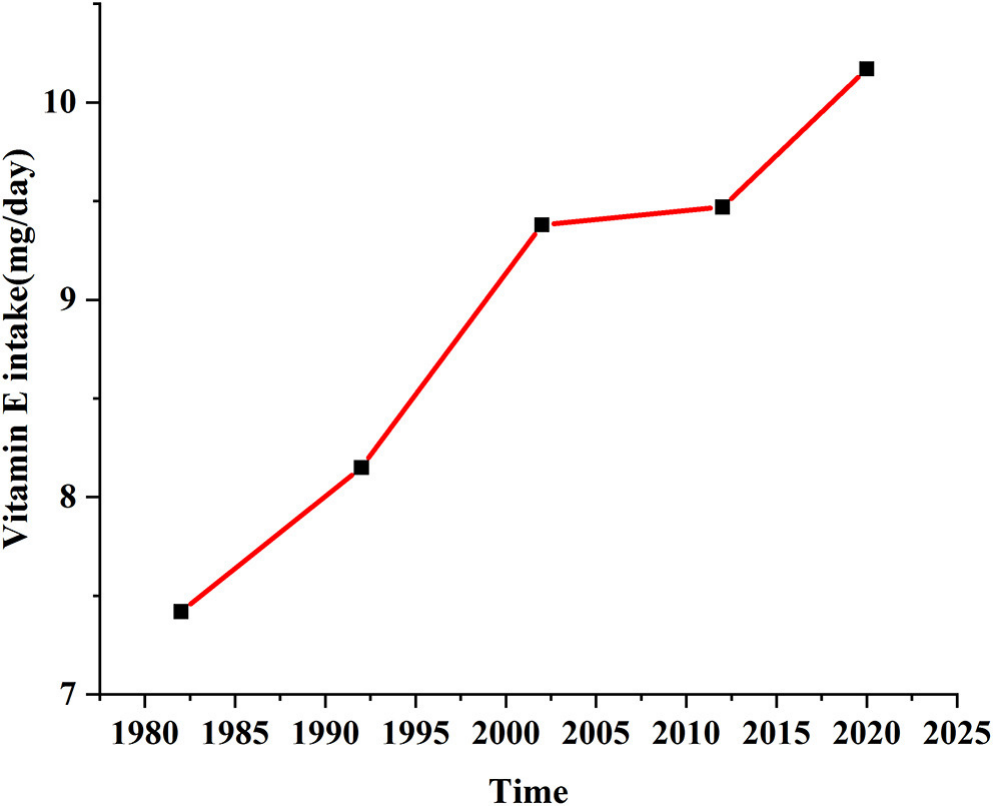
Changes in vitamin E intake of Chinese residents between 1982 and 2020.

The data in the schematic diagram of VE intake of Chinese residents in the past 40 years mentioned in Figure 1 are from the Chinese food composition table, the monitoring report on the nutrition and health status of Chinese residents, the outline of food and nutrition development in China, the 1992 national nutrition survey, and the 2001–2012 monitoring of nutrition and health of Chinese residents. Based on the consumption of vegetable oil in 1982, 1992, 2002, 2012, and 2020, and then combined with the contents of VE in vegetable oils, the VE intake of Chinese residents from vegetable oils in each year was calculated.

## Results

### Contents and Compositions of Tocopherols in Different Foods

VE is synthesized only in photosynthetic organisms, including higher plants. A balanced dietary pattern for Chinese residents covers several major categories of essential basic foods, including cereal, vegetable, fruit, livestock, poultry, fish, egg, milk, bean, nut, and cooking oils. Since the contents of tocotrienols in foods are usually low compared with those of tocopherols and do not affect the assessment of VE dietary intake (18). Therefore, we selected the tocopherols for the calculation. In this study, the contents of tocopherols in 12 kinds of major foods including cereal, grain, potato, bean, vegetable, fruit, nut, vegetable oil, meat, egg, milk, and aquatic products were collected and shown in Supplementary Tables 1, 2. The highest content of tocopherols was found in vegetable oils, such as sunflower oil. Vegetable oils mainly contain α-tocopherol and γ-tocopherol, as their main source of antioxidants (19). This is followed by nuts, with a weighted average tocopherol content of 165.53 mg kg^−1^. Some studies have shown that the oils extracted from walnuts, almonds, peanuts, hazelnuts, and macadamia nuts are a good source of tocopherols (20, 21). Meanwhile, cereal grains, eggs, and aquatic products also contain some amount of tocopherols. The tocopherol content of different types of grains varies greatly. As shown in Supplementary Table 1, wheat has the higher VE content than rice. Although the amount of tocopherols in fish varies with different species, α-tocopherol is the predominant form of VE (22).

VE was first discovered in green leafy vegetables in 1922, and it was found to be an essential nutrient for plant reproduction (23). However, vegetables, fruits, and dairy products were found to have lower levels of tocopherols compared to vegetable oils and nuts. Cruciferous vegetables are a good source of antioxidants, and the antioxidant content varies considerably both within and between subspecies (24, 25).

Palm, soybean, rapeseed, sunflower seed, corn, and coconut oil are the most exploited vegetable oils worldwide, which account for 93% consumption of vegetable oils (26). In China, peanut oil, sesame oil, flaxseed oil, Camellia oil, cottonseed oil, olive oil, and grape seed oil are also important source to intake VE. So, as shown in Supplementary Table 2, composition and content of tocopherols in 12 kinds of vegetable oils were collected and analyzed in this study. The tocopherols in vegetable oils are mainly α-, γ-, and δ-tocopherols. The content and composition of tocopherols in vegetable oils are presented in Supplementary Table 2. Soybean oil had the highest total tocopherol content of 930.80 mg kg^−1^, high γ-tocopherol and δ-tocopherol content, and very low α-tocopherol content. Palm oil is very low in tocopherols, with the total tocopherol content of 152.40 mg kg^−1^, and is mainly produced in Malaysia and Indonesia. The total content of tocopherols in rapeseed oil is 608.90 mg kg^−1^, and the main components are α-tocopherol and γ-tocopherol. The types of tocopherols in corn oil and rapeseed oil are similar, mainly containing α- and γ-tocopherols, with a total tocopherol content of 509.40 mg kg^−1^. Sunflower oil ranks fourth in consumption after soybean oil, rapeseed oil, and palm oil, with a total tocopherol content of 546.00 mg kg^−1^. The total tocopherol content of peanut oil was 420.60 mg kg^−1^ and mainly contained α and γ species. Olive oil is known in the West as “liquid gold.” The total tocopherol content in olive oil is 169.10 mg/kg, with the high content of α-tocopherol, accounting for 85% of the total tocopherol content. Moreover, sesame oil is a traditional Chinese flavored vegetable oil, and the total content of tocopherols in sesame oil is 685.30 mg kg^−1^ (mainly consisting of γ-tocopherol). Flaxseed oil and cottonseed oil include mainly γ-tocopherol (27).

### Assessment of Dietary Intake of VE in the Chinese Population

Combining the tocopherol levels of vegetable oils and other foods, we reviewed the tocopherol levels of a total of 10 food items, excluding vegetable oils, covering almost all food groups in the daily diet of the Chinese population, including cereals, potatoes, legumes, vegetables, fruits, nuts, meat, eggs, dairy products, and aquatic products. For food with low consumption frequency and consumption, their impact on the assessment of the overall VE intake of the Chinese population was minimal. α-Tocopherol has a very important antioxidant effect on cell membranes (28) and is also more effective in improving oxidative stability and reducing the relative oxidation rate with increasing temperature (29). In this study, we used the total content of tocopherols and the α-TE to assessment of dietary intake of VE in the Chinese population.

The contributions of different foods and vegetable oils to the VE intake of the Chinese population are listed in Tables 1, 2, respectively. The total VE intake (calculated by α-TE) from the daily diet of the residents was 10.17 mg day^−1^, while the recommended dietary reference intake (AI) of VE for adults in China is 14.0 mg day^−1^. Comparing the results, the actual VE intake of Chinese residents did not meet the recommended intake. The contribution of vegetable oil to VE intake ranked first in the dietary intake of residents, accounting for 46.76% of VE intake (4.76 mg day^−1^), while cereals ranked second with 18.24% (1.85 mg day^−1^), followed by vegetables at 9.42% (0.96 mg day^−1^). The highest contributing vegetable oil was rapeseed oil, accounting for 10.73% (1.09 mg day^−1^) of the total VE intake. Although soybean oil had the highest total tocopherol content, its lower α-tocopherol content meant that its contribution toward dietary intake was lower than that of rapeseed oil.

**Table 1:** Contribution of commonly consumed foods to vitamin E dietary intake.

**Food categories**	**Consumption (g/day)**	**α-TE (mg/kg)**	**Total tocopherol content (mg/kg)**	**Intake of vitamin E (mg/day)**	**Percent of total intake of vitamin E (%)**
Cereal	377.00	4.92	9.90	1.85	18.45
Coarse cereals	28.00	7.49	21.73	0.21	2.09
Potato	36.00	1.86	3.10	0.07	0.67
Bean	11.00	21.29	183.49	0.23	2.33
Vegetable	269.00	3.56	5.55	0.96	9.53
Fruit	41.00	4.66	8.20	0.19	1.90
Nut	4.00	50.08	165.53	0.20	1.99
Vegetable oil	37.00	128.55	642.01	4.76	46.76
Meat	104.00	6.15	8.71	0.64	6.36
Egg	33.00	12.68	19.48	0.42	4.16
Milk	66.00	1.38	1.70	0.09	0.90
Aquatic products	45.00	12.25	22.35	0.55	5.48
Total				10.17	100.00

*Data on consumption of various types of foods are from the United States Department of Agriculture (USDA), the 2020 China Statistical Yearbook, and the China Population Nutrition and Health Status Monitoring Report*.

**Table 2:** Contribution of vegetable oils to vitamin E dietary intake.

**Vegetable oil type**	**Tocopherol content (mg/kg)**	**Consumption ratio (%)**	**Vitamin E intake (mg/day)**	**Contribution of vitamin E (%)**
Soybean oil	61.10	43.00	0.97	9.56
Rapeseed oil	147.50	20.00	1.09	10.73
Palm oil	128.82	17.00	0.81	7.97
Peanut oil	194.34	8.00	0.58	5.66
Sunflower oil	450.83	6.00	1.00	9.84
Other oils	138.03	6.00	0.31	3.01

Though traditional VE dietary intake assessment was conducted by using α-tocopherol equivalents, other tocopherols show various functions different from α-Tocopherol. For example, γ-Tocopherol is found to be a more potent free radical scavenger than α-tocopherol *in vitro* (30). Therefore, we performed VE dietary intake assessment by using the total content of tocopherols. The results were shown in Supplementary Table 3. The VE intake of Chinese residents was 35.38 mg day^−1^ per capita. Among the 12 kinds of foods, vegetable oils contributed the most (>50%), followed by cereals. These results were almost consistent with those of the α-TE.

## Discussion

### Estimated Dietary VE Intake of Residents in Different Dietary Pattern of China

China is a large geographical area, and residents of different regions have different dietary habits. The different kinds of cereals and edible oils are the main differences of dietary habits for residents of different regions in China. Therefore, we selected several regions in China with different dietary structures to analyze and compare VE dietary intake. In this section, the estimated VE intake from cereals or edible oils was calculated by product of α-TE value of a particular kind of edible oil (like rapeseed oil) or cereal (like wheat) and the daily consumption of edible oils or cereals queried in the USDA and China Statistical Yearbook. The results are presented in Table 3.

**Table 3:** Estimated vitamin E intake of residents in different dietary pattern of China.

**Dietary pattern**	**Classic region**	**Vitamin E intake (mg/day)**
Soybean oil+ Wheat	Inner Mongolia, Hebei, Shanxi	7.81
Soybean oil+ Rice	Heilongjiang, Jilin, Liaoning	6.79
Rapeseed oil+ Wheat	Qinghai	11.04
Rapeseed oil+ Rice	Hubei, Hunan, Shanghai, Zhejiang, Jiangsu, Anhui, Jiangxi, Sichuan	10.02
Peanut oil+ Wheat	Henan, Shandong, Anhui	12.77
Peanut oil+ Rice	Guangdong, Guangxi, Hong Kong, Macao	11.75
Camellia oil+ Rice	Camellia Oil Belt from Yunnan to Zhejiang	5.54
Sunflower seed Oil+ Wheat	Xinjiang	22.26
Soybean oil+ Indica rice	Taiwan	7.09
Butter +Highland barely	Tibet	6.80

The distribution of edible oil varieties in China shows obvious geographical differences. The northwestern region such as Gansu province is vast in area but also arid and water-scarce, with fragile ecological conditions. The Qinghai and Tibetan regions are the main settlements of Tibetans, and the region has very little land suitable for cultivation, with its alpine climate. The main crop in the region is barley. So, the daily diet in this region has obvious regional characteristics. The vitamin sources available to the residents in this region mainly come from barley and ghee. The VE intake of the residents in this region (6.80 mg day^−1^) is lower than the national average, indicating serious VE deficiency.

The northeastern China's Heilongjiang, Jilin, and Liaoning province have a dietary structure consisting of soybean oil and rice. Thus, VE supplementation is essential for residents of these areas. Meanwhile, the tocopherols were obtained primarily from soybean oil and wheat and also showed insufficient VE intake (7.81 mg day^−1^) for residents in Hebei, Shanxi, Shaanxi and Inner Mongolia. Residents in Henan, Shandong, Anhui, Guangxi, Guangdong, Hong Kong, and Macao had relatively high consumption rates of peanut oil, while those in South China had a sufficient intake of fresh vegetables and aquatic products rich in VE. Residents in areas in the Yangtze River basin such as Hubei and Hunan mainly consumed rapeseed oil, and the VE content of both peanut oil and rapeseed oil is relatively high. Edible oils in Shanghai, Zhejiang, Jiangsu, Anhui, and Jiangxi are also dominated by rapeseed oil, and the intake of VE of residents in these areas is 10.02 mg day^−1^, which is close to the national average. However, since the VE content of tea oil is lower, while the tocopherol content of rice is also lower compared to wheat and other grains, residents from these regions had the lowest national VE intake of 5.54 mg day^−1^. The geographical conditions in the south are complex, and there are differences in dietary habits and dietary structure in different provinces. This causes the VE intake to vary among residents of different regions in the south. The dietary structure of China has long been influenced by the living environment and is adapted to local conditions. So, the dietary intake structure of residents in different regions varies and results in very different levels of VE in the dietary intake.

In summary, Chinese residents do not consume enough VE. There is no doubt that supplementing VE through food is the healthiest option. Edible oil is an essential part of our daily diet, which not only improves the color of dishes and adds flavor to food but also provides a rich source of VE. Among main edible oils, rapeseed oil, peanut oil, and sunflower oil have high tocopherol content and are a good choice for VE supplementation. Meanwhile, it is necessary to develop some high VE edible oils including *Eucommia ulmoides* Oliver seed oil and sea buckthorn seed oil (31) to improve the dietary intake of VE, especially for residents with low VE intake.

### Historical Dietary Intake of VE by Chinese Residents

Over the past 40 years, the dietary structure of Chinese residents has changed dramatically. In the past, residents received most of their calories from grains and fats, but today, the intake of fruits and vegetables has gradually increased. VE intake from 1982 to 2020 in China are illustrated in Figure 1. The consumption of vegetable oil has gradually increased, and the intake of VE by Chinese residents has also gradually increased. VE intake among Chinese residents has increased year by year since 1982, showing an upward trend. Economic and social development has led to a dramatic improvement in the standard of living of Chinese residents. In just four decades, China has solved the problem of feeding 1.3 billion people and is now dealing with an increasing number of nationals who are overweight, obese, or dealing with nutritional imbalance due to food-borne diseases.

The structure of the food consumption of residents has undergone different changes in various periods. As the income level of the population increased and due to the advancement of industrialization and urbanization, there were two more significant changes in the food consumption of the populace. First, the per capita consumption of cereal products continued to rise until 1984 but exhibited a downward trend thereafter. Second, the consumption of animal products, such as livestock and poultry meat, aquatic products, and dairy products, as well as fruits and vegetables, increased significantly. Although the consumption of fruits and vegetables has increased, it still has not reached the 500 g day^−1^ level recommended in the Dietary Guidelines for Chinese Residents. In recent years, the domestic demand for vegetable oils has been on the rise. Therefore, selecting and breeding high VE oil crops or improving the VE content in edible oil and oilseed products by other means are very important to improve the national dietary health level and achieving improved national physical fitness through the adjustment of dietary structure.

## Conclusion

Overall, the current daily dietary intake of VE by Chinese residents does not meet the needs of the human body. From the calculations in the previous sections, it was concluded that vegetable oils are the largest contributor of VE in a daily diet; so, the choice and quality of vegetable oil varieties affect the VE intake of Chinese residents. Among several bulk vegetable oils consumed daily, soybean oil had the highest total tocopherol content. However, its α-tocopherol content was low, while canola and peanut oils had higher α-TE content and can be used as the main dietary source of VE supplementation. The VE intake of residents in different regions of China was further compared, and significant differences in the intake of VE in different regions were found due to differences in geography and dietary habits. The intake of VE by Chinese residents from 1982 to 2020 was also compared. Over the past 40 years, the dietary structure of Chinese residents has changed dramatically. Their food consumption has become diversified, and the purpose of food intake has changed from satisfying sustenance to maintaining health. The consumption of cereals has declined, and the consumption of fruits and vegetables has risen. Although the dietary nutrition of Chinese residents has improved, there is still much need to intake VE from foods. This study provides with scientific evidence for reasonable VE supplementation.

## Data Availability Statement

The raw data supporting the conclusions of this article will be made available by the authors, without undue reservation.

## Author Contributions

YZ and LZ: methodology and writing—original draft. LZ: conceptualization and writing—review and editing. XQ, XueyW, XuefW, FM, LY, JM, and JJ: formal analysis. PL and LZ: funding acquisition. PL: supervision. All authors contributed to the article and approved the submitted version.

## Funding

This work was supported by the National Key Research and Development Project of China (2021YFD1600101), National Nature Foundation Committee of P.R. China (31871886), the National Major Project for Agro-product Quality & Safety Risk Assessment (GJFP2021002), and the earmarked fund for China Agriculture Research System (CARS-12).

## Conflict of Interest

The authors declare that the research was conducted in the absence of any commercial or financial relationships that could be construed as a potential conflict of interest.

## Publisher's Note

All claims expressed in this article are solely those of the authors and do not necessarily represent those of their affiliated organizations, or those of the publisher, the editors and the reviewers. Any product that may be evaluated in this article, or claim that may be made by its manufacturer, is not guaranteed or endorsed by the publisher.

## Supplementary Material

Supplementary Table 1 Tocopherol contents of main foods except vegetable oils.

Supplementary Table 2 Composition and content of tocopherols in main kinds of vegetable oils.

Supplementary Table 3 The contribution of total tocopherol from main kinds of foods.
