# Supplementary material for: Contribution of Tocopherols in Commonly Consumed Foods to Estimated Tocopherol Intake in the Chinese Diet
Source: Front Nutr. 2022 Jun 8;9:829091. doi: 10.3389/fnut.2022.829091 (PMC9213682; doi:10.3389/fnut.2022.829091)
Supplement: Supplementary Table 1 — Tocopherol contents of main foods except vegetable oils. [file Table_1.docx]

Supplementary Material

# Supplementary Tables

## Table S1 Tocopherol contents of main foods except vegetable oils

## Table S2 Composition and content of tocopherols in main kinds of vegetable oils

Table S3 The contribution of total tocopherol from main kinds of foods

Table S1 Tocopherol contents of main foods except vegetable oils

| Food Categories | Food Name | α-TE content  (mg/kg) | Total tocopherol content  (mg/kg) |
| --- | --- | --- | --- |
| Cereal | Rice | 3.50 | 6.80 |
|  | Wheat | 6.52 | 13.20 |
|  | Coarse cereals | 12.05 | 27.28 |
| Potato | Potato | 0.92 | 3.40 |
|  | Sweet potato | 2.80 | 2.80 |
| Bean | Soybean | 22.85 | 189 |
|  | Mung bean | 10.69 | 109.50 |
|  | Red bean | 6.85 | 143.60 |
| Vegetable | Carrot | 3.10 | 3.10 |
|  | Long bean | 3.47 | 23.80 |
|  | Kidney bean | 4.86 | 12.40 |
|  | Cowpea | 0.18 | 6.50 |
|  | Eggplant | 11.30 | 11.30 |
|  | Red pepper | 3.77 | 4.40 |
|  | Tomato | 2.76 | 4.20 |
|  | Green pepper | 3.80 | 3.80 |
|  | Sweet pepper | 4.10 | 4.10 |
|  | Long crooked squash | 0.03 | 0.30 |
|  | Cucumber | 1.06 | 4.90 |
|  | Balsam pear | 6.34 | 8.50 |
|  | Pumpkin | 2.97 | 3.60 |
|  | Summer squash | 3.40 | 3.40 |
|  | Wax gourd | 0.44 | 0.80 |
|  | Chinese chives | 4.26 | 5.70 |
|  | Chinese cabbage | 3.60 | 3.60 |
|  | Cabbage | 2.32 | 5.00 |
|  | Leaf mustard | 6.41 | 7.40 |
|  | Broccoli | 6.07 | 7.60 |
|  | Spinage | 14.88 | 17.40 |
|  | Coriander | 6.92 | 8.00 |
|  | Garland chrysanthemum | 4.94 | 9.20 |
|  | Asparagus lettuce | 0.89 | 2.40 |
|  | Lettuce | 4.50 | 4.50 |
|  | Bamboo shoots | 0.32 | 0.50 |
| Fruits | Apple | 2.43 | 3.70 |
|  | Banana | 2.64 | 4.80 |
|  | Pear | 3.89 | 11.20 |
|  | Grape | 3.98 | 10.90 |
|  | Peach | 2.99 | 9.00 |
|  | Tangerine | 12.38 | 15.80 |
|  | Orange | 5.15 | 5.60 |
|  | Grapefruit | 1.70 | 1.70 |
|  | Cherry | 4.52 | 22.20 |
| Nuts | Almond | 249.92 | 271.70 |
|  | Pistachio | 28.13 | 193.60 |
|  | Walnut | 21.23 | 143.50 |
| Meats | Beef | 4.91 | 6.80 |
|  | Chicken | 13.78 | 18.10 |
|  | Pork | 4.27 | 6.70 |
|  | Mutton | 4.80 | 4.80 |
| Eggs | Egg | 7.37 | 13.50 |
|  | Duck eggs | 41.16 | 49.80 |
|  | Goose egg | 36.63 | 45.00 |
|  | Quail egg | 17.95 | 30.80 |
| Milk | Milk | 0.93 | 1.30 |
|  | Whole milk powder | 4.80 | 4.80 |
| Aquatic Products | Fish | 20.30 | 20.30 |
|  | Shrimp and Crab diver | 17.89 | 23.38 |
|  | Shellfish | 30.44 | 72.10 |
|  | Algae | 24.47 | 31.40 |

Table S2 Composition and content of tocopherols in main kinds of vegetable oils

| Types of oil | α-Tocopherol  (mg/kg) | β+γ-Tocopherol  (mg/kg) | δ-Tocopherol  (mg/kg) | α-TE  (mg/kg) | Total tocopherol content（mg/kg） |
| --- | --- | --- | --- | --- | --- |
| Soybean oil | Tr | 575.50 | 355.30 | 61.10 | 930.80 |
| Rapeseed oil | 108.10 | 382.10 | 118.70 | 147.50 | 608.90 |
| Palm oil | 126.20 | 26.20 | 0.00 | 128.82 | 152.40 |
| Peanut oil | 174.50 | 193.10 | 53.00 | 194.34 | 420.60 |
| Sunflower seed oil | 383.50 | 134.10 | 28.40 | 397.19 | 546.00 |
| Corn oil | 144.20 | 351.30 | 13.90 | 179.47 | 509.40 |
| Sesame oil | 17.70 | 646.50 | 21.10 | 82.56 | 685.30 |
| Cottonseed oil | 193.10 | 671.40 | Tr | 260.24 | 864.50 |
| Olive oil | 143.80 | 22.40 | 2.90 | 146.07 | 169.10 |
| Grape seed oil | 189.80 | 90.10 | 12.30 | 198.93 | 292.20 |
| Flaxseed oil | 34.20 | 382.30 | 12.60 | 72.56 | 429.10 |
| Camellia oil | 14.50 | 103.00 | 161.50 | 26.42 | 279.00 |

Table S3 Contribution of total tocopherol from main kinds of foods

| Food Categories | Consumption  (g/day) | Total tocopherol content  （mg/kg） | Intake of vitamin E  (mg/day) | Percent of Total Intake of vitamin E  (%) |
| --- | --- | --- | --- | --- |
| Cereal | 377.00 | 9.90 | 3.73 | 10.55 |
| Coarse cereals | 28.00 | 21.73 | 0.61 | 1.72 |
| Potatoes | 36.00 | 3.10 | 0.11 | 0.32 |
| Beans | 11.00 | 183.49 | 2.02 | 5.70 |
| Vegetables | 269.00 | 5.55 | 1.49 | 4.22 |
| Fruits | 41.00 | 8.20 | 0.34 | 0.95 |
| Nuts | 4.00 | 165.53 | 0.66 | 1.87 |
| Vegetable oil | 37.00 | 642.01 | 23.75 | 67.13 |
| Meats | 104.00 | 8.71 | 0.91 | 2.56 |
| Eggs | 33.00 | 19.48 | 0.64 | 1.82 |
| Milk | 66.00 | 1.70 | 0.11 | 0.32 |
| Aquatic Products | 45.00 | 22.35 | 1.01 | 2.84 |
| Total |  |  | 35.38 | 100.00 |
